# Editorial

**Published:** 1898-09

**Authors:** 


### Editorial.

### HOSPITALS AND HOSPITALISM.

Dr. George M. Gould never coined a better word than hospitalism. In his address before the American Academy of Medicine, he shows the outcome of the unlimited facility for the treatment of the sick and wounded in hospitals. The abuse of a good thing often makes the use of that thing more apparent. The hospitals of our country are to be used for the care of the needy, sick, and wounded, or for the accommodation of the patients of all classes and conditions. Even incidentally the care of the doctors should be only for the sake of their patients. But the word hospitalism now really embraces the abuse of the office and object of the visiting staff as well as the direct treatment of patients. These reflections are eminently appropriate in view of the embroglio of the New York hospitals. It appears that the New York hospitals are in danger of drifting into a special grade of hospitalism. This may be called college hospitalism. Beginning with the woman's hospital founded by the great J. Marion Sims, the term has been applicable also to Bellevue Hospital.

It is known that Dr. Sims was removed from this hospital by the desire of others for the position; and that a short while ago as many as twenty-seven physicians were removed from a New York hospital because they had no position in the Medical College.

The fact that such even as Sims, McDowell, B. O. Baldwin, Jerome Cochrane, and others, rose in spite of no hospital and college connection should be sufficient evidence of the great advantages for observation in private practice. But what had become of the code of ethics of the American Medical Association, of the *jusjurandum* of Hippocrates, or of the innate feeling of the eternal fitness of things when the selfish ambition of men led them to political methods even in the management of the sick?

